# Al-induced CsUGT84J2 enhances flavonol and auxin accumulation to promote root growth in tea plants

**DOI:** 10.1093/hr/uhad095

**Published:** 2023-05-05

**Authors:** Xiaolan Jiang, Sanyan Lai, Dexu Kong, Xiaohan Hou, Yufeng Shi, Zhouping Fu, Yajun Liu, Liping Gao, Tao Xia

**Affiliations:** State Key Laboratory of Tea Plant Biology and Utilization, Anhui Agricultural University, Hefei, Anhui, China; State Key Laboratory of Tea Plant Biology and Utilization, Anhui Agricultural University, Hefei, Anhui, China; State Key Laboratory of Tea Plant Biology and Utilization, Anhui Agricultural University, Hefei, Anhui, China; State Key Laboratory of Tea Plant Biology and Utilization, Anhui Agricultural University, Hefei, Anhui, China; State Key Laboratory of Tea Plant Biology and Utilization, Anhui Agricultural University, Hefei, Anhui, China; State Key Laboratory of Tea Plant Biology and Utilization, Anhui Agricultural University, Hefei, Anhui, China; School of Life Science, Anhui Agricultural University, Hefei, Anhui, China; School of Life Science, Anhui Agricultural University, Hefei, Anhui, China; State Key Laboratory of Tea Plant Biology and Utilization, Anhui Agricultural University, Hefei, Anhui, China

## Abstract

Although Al is not necessary or even toxic to most plants, it is beneficial for the growth of tea plants. However, the mechanism through which Al promotes root growth in tea plants remains unclear. In the present study, we found that flavonol glycoside levels in tea roots increased following Al treatment, and the Al-induced UDP glycosyltransferase CsUGT84J2 was involved in this mechanism. Enzyme activity assays revealed that rCsUGT84J2 exhibited catalytic activity on multiple types of substrates, including phenolic acids, flavonols, and auxins *in vitro*. Furthermore, metabolic analysis with UPLC-QqQ-MS/MS revealed significantly increased flavonol and auxin glycoside accumulation in *CsUGT84J2*-overexpressing *Arabidopsis thaliana*. In addition, the expression of genes involved in the flavonol pathway as well as in the auxin metabolism, transport, and signaling pathways was remarkably enhanced*.* Additionally, lateral root growth and exogenous Al stress tolerance were significantly improved in transgenic *A. thaliana*. Moreover, gene expression and metabolic accumulation related to phenolic acids, flavonols, and auxin were upregulated in *CsUGT84J2*-overexpressing tea plants but downregulated in *CsUGT84J2*-silenced tea plants. In conclusion, Al treatment induced *CsUGT84J2* expression, mediated flavonol and auxin glycosylation, and regulated endogenous auxin homeostasis in tea roots, thereby promoting the growth of tea plants. Our findings lay the foundation for studying the precise mechanisms through which Al promotes the growth of tea plants.

## Introduction

In acidic soils (pH < 5), Al is decomposed into soluble Al ions, which can be easily absorbed by plants, leading to toxic effects in most plants and significant decrease in crop yield [1–4]. Major plant mechanisms of Al tolerance include exclusion and tolerance. As such, the mechanism of Al exclusion involves the secretion of organic acids and phenolics, while the mechanism of Al tolerance primarily involves the modification of cell wall, complexation of organic acids and phenolics, and transportation of Al [4–6].

Unlike most crops, tea plants are Al-tolerant and can adapt to higher free Al ion levels in the soil. Even moderate Al concentrations can markedly promote the growth and development of tea plants, although excess Al may lead to toxicity [6–8]. Recent research on the Al tolerance of tea plants was mainly focused on the complexation of organic acids and modification of cell walls. For instance, according to Martin *et al.* [9], oxalic acid complexes play pivotal roles in the Al tolerance mechanisms of tea roots. Moreover, the Al tolerance of tea plants is related to the methyl-esterification of pectin and organic acids in the root tip cell wall [10]. Low-concentration Al treatment affected the activity of genes and enzymes controlling pectin and hemicellulose synthesis, thereby loosening the cell wall and promoting root elongation in seedlings [11]. In the absence of Al^3+^ ions, however, cell differentiation in the root meristem was rapidly suppressed, leading to prompt cessation of root growth [7]. However, the mechanisms through which Al promotes tea plant growth remain unclear (Fig. S1, see online supplementary material).

Auxin is a key regulator of almost all aspects of plant development, including morphogenesis and adaptive response [12]. Plant development from embryos to flowers depends on the formation of local auxin gradients, which are established and maintained through close regulatory interactions among metabolism, signaling, and transportation [1, 3, 13]. Auxin is mainly biosynthesized in young tissues and distributed to other plant parts via polar transport. Endogenous plant flavonoids may be the natural modulators of auxin efflux and polar transport [14]. Flavonoids are seen as auxin transport inhibitor in the apical buds and root tip. Flavonols, such as 7-Rhamnosylated flavonols, negatively altered the polar transport of auxin. Flavonoids can bind and inhibit the auxin transport proteins ABCB1 and ABCB19 as well as interact with hormones to affect plant growth and development [15–17]. In addition, flavonols have been reported to mediate stabilization of PIN efflux complexes, thus redirecting polar auxin streams [18].

In plants, glycosylation is a common biochemical process of great significance [19]. It transfers donor sugar molecules or related groups to specific acceptors by generating glycosidic bonds, thereby altering the solubility, stability, and toxicity potential of the corresponding aglycons [20]. Glycosylation plays vital roles in plant seed germination, seedling growth, reproduction, stress response, and other life processes [21]. In addition, auxin glycosylation is an important mechanism for auxin inactivation and is involved in auxin transport [22, 23]. In transgenic lines accumulating 1-*O*-IAGlc, indole-3-acetic acid (IAA) levels are controlled through a complex tissue-specific process [14].

To date, several plant auxin glycosyltransferases have been identified. In particular, *AtUGT74E2* is an auxin glucosyltransferase that preferentially uses indole-3-butyric acid (IBA) as the substrate. Because IBA and IAA transform each other and co-regulate the overall auxin homeostasis, IBA glucosyltransferase overexpression disrupts the overall auxin homeostasis and alters plant morphology [24]. Meanwhile, *AtUGT74D1* can effectively glycosylate and modify natural plant auxins, such as IBA and IAA, and affect plant growth and development. As such, plants overexpressing *AtUGT74D1* have been reported to exhibit curled leaves and reduced leaf petiole angles [25]. In addition, *AtUGT84B1* is an auxin glycosyltransferase that acts on IAA and phenylacetic acid as the substrate. In plants overexpressing *AtUGT84B1*, the leaves are round, wrinkled, and curled and their midrib is destroyed [26]. Taken together, these findings suggest that auxin glycosyltransferases play critical roles in auxin activity and plant development.

In general, tea plants are Al-tolerant. Low Al concentrations can promote the growth of tea plants, although the underlying regulatory mechanism remains obscure. In the present study, we screened for an Al-induced glycosyltransferase (*CsUGT84J2*) associated with flavonol glycosides and plant hormones. Its gene function was verified using *in vitro* enzyme activity assays, heterologous expression in *Arabidopsis thaliana*, and transient expression in tea plants. Moreover, changes in the related metabolites and gene expression patterns in *CsUGT84J2*-overexpressing and *CsUGT84J2-*silenced plants were analysed. Additionally, the Al tolerance of *CsUGT84J2*-overexpressing *A. thaliana* was investigated. Our study lays the foundation for research on the precise mechanisms through which Al promotes the growth of tea plants.

## Results

### Identification of an Al-induced UGT (*CsUGT84J2*) in tea roots

**Figure 1:**
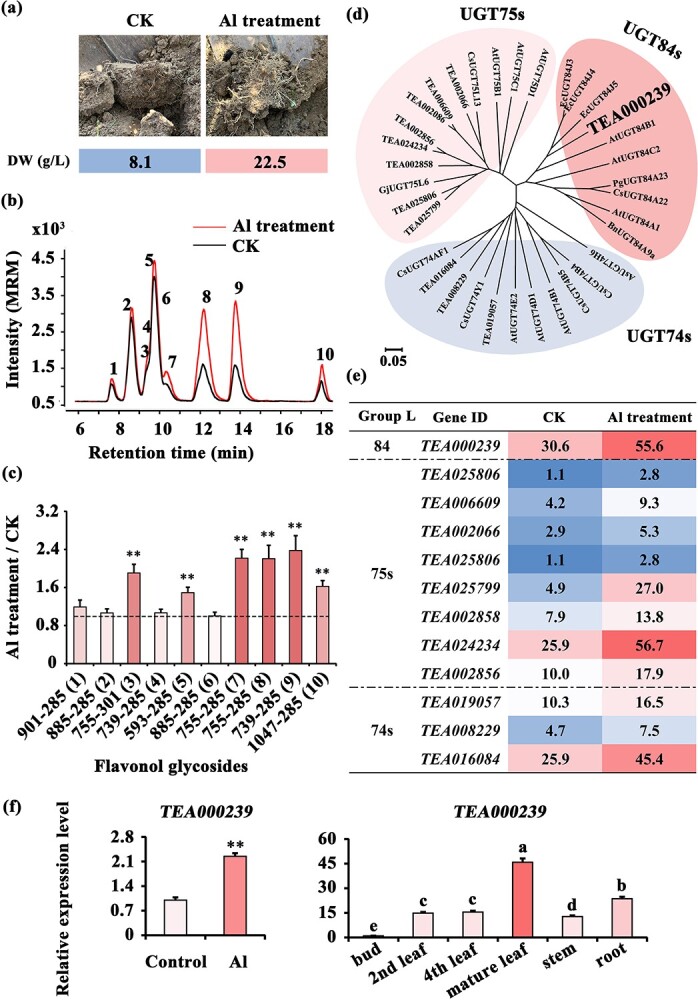
Identification of an Al-induced UGT in tea plants. **a**, Phenotype of roots in tea plantations treated with Al. **b**, Mass spectrum in the MRM mode of flavonol glycosides in tea roots under Al and control treatments. **c**, Changes in flavonol glycoside accumulation in tea roots under Al and control treatments. **d**, Phylogenetic analysis of 12 Al-induced UGTs in the L group. **e**, Expression patterns of the corresponding genes under Al treatment. **f**, Gene expression of *CsUGT84J2* under Al treatment and in different organs analysed by qRT-PCR.

Many studies have reported that Al can promote the growth of tea plants. In the studied tea plantation, the root weight per unit volume of soil was almost two times enhanced following Al treatment (Fig. 1a). However, the mechanism through which Al promotes root growth in tea plants remains unclear. Because flavonol metabolism affects root growth, in order to study the relationship between flavonol metabolism and the root growth of tea plant, the metabolic profile of tea roots following Al treatment was analysed using UPLC-TOF-MS and UPLC-QqQ-MS/MS. The mass spectrum showed that flavonol glycoside accumulation was markedly increased after Al treatment (Fig. 1b and c, Table S7). In particular, for peaks 8 (*m*/*z* 755–285), 9 (*m*/*z* 739–285), 10 (*m*/*z* 755–285), and 11 (*m*/*z* 739–285), kaempferol glycoside accumulation was more than two times that for the control peak (Fig. 1c).

To assess whether flavonol glycoside accumulation affected the growth of tea roots after Al treatment, based on gene expression levels, we screened out 51 UGTs that responded to Al induction from the transcriptome of Al-treated tea roots. Phylogenetic analysis of these 51 UGTs with those from other functionally identified plants showed that they could be divided into 12 groups, from A to O (Figs S2 and S3**,**Table S5, see online supplementary material). Among these, 12 UGTs belonging to the L group were screened out; the proteins belonging to this group have been reported to be closely related to the glycosylation of benzoates [27], auxins [13, 26, 28–30], and flavonols [31]. The L group could be divided into UGT74, UGT75, and UGT84 subclades (Fig. 1d).

The expression patterns of the 12 identified UGTs after Al treatment were analysed using RNA-Seq. The result of FPKM value showed that all these UGTs were Al-induced, including one in the UGT84 subclade, eight in the UGT75 subclade, and three in the UGT74 subclade (Fig. 1e). TEA000239 and CsUGT84A22 belonged to the UGT84 subgroup (Fig. 1). Both RNA-Seq and qRT-PCR analysis revealed that TEA000239 expression in tea roots under Al treatment was two times that under control treatment (Figs 1e and f). TEA000239 was relatively highly expressed in tea roots, with the FPKM value ranging from 30.6 to 55.6 (Fig. 1e). Among the different tissues and organs, TEA000239 expression was the highest in mature leaves, followed by roots, and the lowest in young leaves (Fig. 1f). Overall, TEA000239 is an Al-induced UGT with high expression in roots.

### *In vitro* enzyme activity of *CsUGT84J2* expressed in *Escherichia coli*

To compare the biological functions of TEA000239, which was designated *CsUGT84J2* (GenBank: KP682363.1), ORFs of the two UGT84 subclades in tea plants, namely *CsUGT84J2* and *CsUGT84A22*, were cloned into the pMAL-c2X vector and successfully transformed into *E. coli* BL21 to induce recombinant enzymes (Fig. S4, see online supplementary material). Following induction and affinity purification, SDS-PAGE suggested that the recombinant proteins were 90–100 kDa in size (Fig. S5, see online supplementary material). Several previous studies have reported that L group UGTs can catalyze the glycosylation of benzoates [27, 32, 33], flavonols [31], or auxins [28, 34, 35]. Therefore, the recombinant CsUGT84J2 (rCsUGT84J2) and rCsUGT84A22 were incubated with benzoate, flavonol, and auxin substrates.

**Figure 2:**
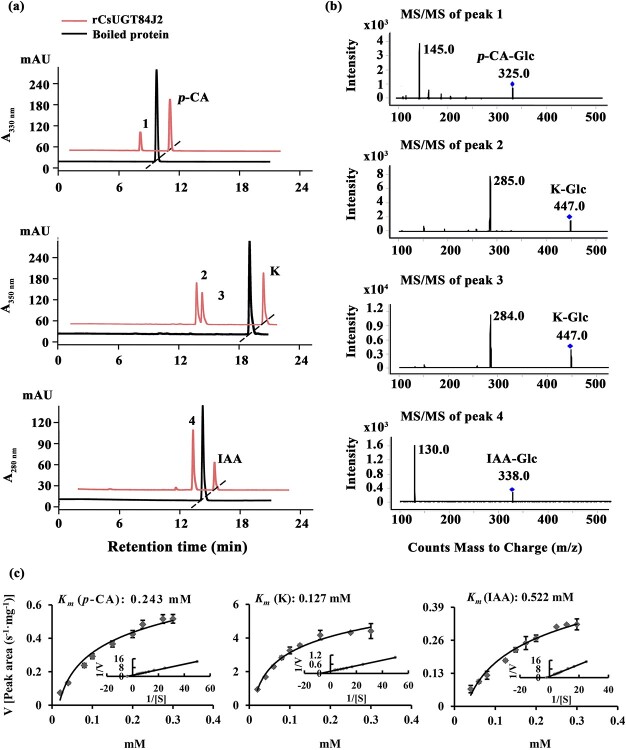
UPLC and UPLC-QqQ-MS/MS analyses of the enzymatic products of rCsUGT84J2. **a**, UPLC analysis of reaction products obtained from rCsUGT84J2 using *p*-coumaric acid, kaempferol, and IAA as substrates. **b**, UPLC-QqQ-MS/MS analysis of the four enzymatic reaction products. **c**, HPLC analysis of the enzyme kinetics of rCsUGT84J2 using *p*-coumaric acid, kaempferol, and IAA as substrates.

UPLC analysis at 330 nm showed that the rCsUGT84J2 protein could catalyze *p*-coumaric acid to produce the corresponding glucoside product (Fig. 2a). Peak 1 was identified by comparing its retention time and UV spectrum with the *p*-CA-Glc standard and by detecting deprotonated ions [M–H]^−^ at *m/z* values of MS 324.9 and MS/MS 145 (Fig. 2b and Table S6). Using other phenylpropionic acids, such as sinapic acid, as the substrate, rCsUGT84J2 could also produce the corresponding sinapic acid glucoside. However, rCsUGT84A22 could not use benzoic acids, such as gallic acid, as sugar acceptors (Table 1). Meanwhile, rCsUGT84A22 could use both benzoic acid and phenylpropionic acid as sugar acceptors (Table 1). UPLC and MS analyses revealed that rCsUGT84J2 proteins catalyzed kaempferol to produce two products (Fig. 2a and b). Peak 2 was identified as K-7-*O*-Glc. Peak 3 was another K-Glc, but its glycosylation site warranted further identification. Using quercetin as the substrate, rCsUGT84J2 produced the corresponding quercetin glucoside. However, rCsUGT84A2 showed no such function (Table 1).

**Table 1:** Enzymatic analysis of the rCsUGT84J2 and rCsUGT84A22 proteins with different substrates.

			**Activity**		
**Sugar donor**	**Sugar acceptor**		**UGT84A22**	**UGT84J2**	**Product**
		Gallic acid	+	−	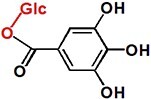
	Benzoic acid	*p*-Hydroxybenzoic acid	+	−	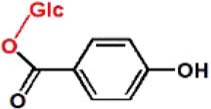
		*p*-Coumaric acid	+	+	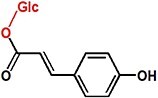
	Phenylpropionic acid	Sinapic acid	+	+	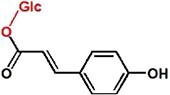
UDP-Glc		Kaempferol	−	+	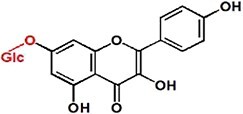
	Flavonols	Quercetin	−	+	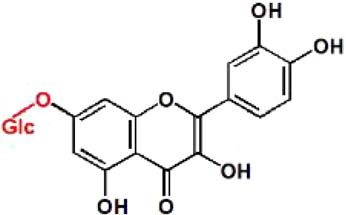
		Indole-3-acetic acid	−	+	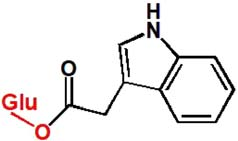
	Auxins	Indole-3-butyric acid	−	+	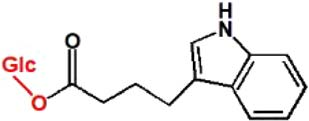
UDP-Rha	Flavonols	Kaempferol	−	+	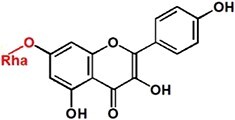

Note: ‘+’: Enzyme activity detected.

‘−’: No enzyme activity detected.

Moreover, except for phenolic compounds, rCsUGT84J2 proteins could catalyze the glycosylation of auxins, such as IAA and IBA (Fig. 2 and Table 1). However, rCsUGT84A22 could not use auxins as sugar acceptors (Table 1). In addition, UDP-Rha could act as a sugar donor for the rCUGT84J2 protein, in addition to UDP-Glc, when the sugar acceptor was a flavonol (Table 1; Fig. S6, see online supplementary material). In summary, enzymatic activity assays showed that rCsUGT84J2 could convert phenylpropionic acid, flavonols, and auxins to their corresponding glucosides, but rCsUGT84A2 could only use benzoates, including benzoic and phenylpropionic acids, as sugar acceptors (Fig. 2).

To characterize the biochemical properties of rCsUGT84J2, kaempferol, *p*-coumarc acid, and IAA were used as substrates to determine the optimal reaction temperature and pH. When kaempferol and *p*-coumaric acid were used as sugar acceptors, the enzymatic activity of rCsUGT84J2 increased gradually from 25°C to 40°C and then slightly declined from 40°C to 45°C (Fig. S7a, see online supplementary material). Therefore, 40°C was the optimal temperature when kaempferol and *p*-coumaric acid were used as substrates. However, for IAA, the optimal temperature was 30°C, which was much lower than that for phenolic compounds (Fig. S7a, see online supplementary material). From pH 7 to 9.5, K-7-*O*-Glc accumulated gradually, but from pH 9.5 to 11, the accumulated product declined (Fig. S7b, see online supplementary material). Therefore, pH 9.5 was the optimal reaction pH when kaempferol was used as the sugar acceptor. Moreover, *p*-CA-Glc and IAA-Glc accumulation increased from pH 5 to 6.5 and declined from pH 6.5 to 9.5; thus, pH 6.5 was the optimal reaction pH when *p*-coumaric acid and IAA were used as sugar acceptors (Fig. S7b, see online supplementary material).

The kinetics of rCUGT84J2 were further investigated under optimal enzyme reaction conditions. rCUGT84J2 displayed the highest affinity for kaempferol (*K*_M_ = 0.127 μM), followed by *p*-coumaric acid (*K*_M_ = 0.243 μM) and IAA (*K*_M_ = 0.522 μM) (Fig. 2c). Therefore, based on kinetic parameters, the catalytic activity of rCsUGT84J2 on kaempferol was higher than that on *p*-coumaric acid and IAA.

### Heterologous expression of *CsUGT84J2* in *A. thaliana*

**Figure 3:**
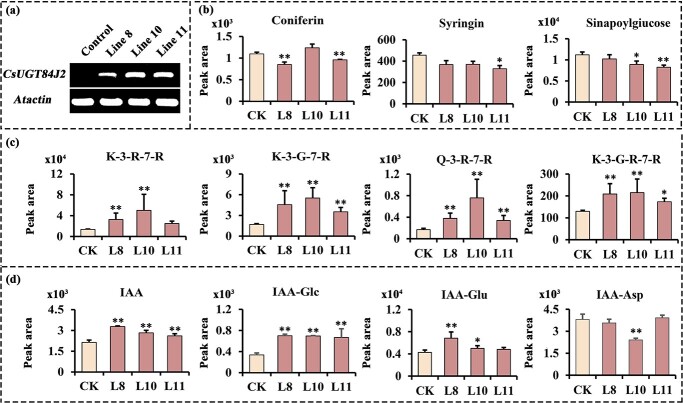
Metabolic analysis of phenolic acids, flavonols, and auxins in *CsUGT84J2-*overexpressing *Arabidopsis thaliana.***a**, Steady-state levels of *CsUGT84J2* transcripts in transgenic homozygous *Arabidopsis* lines. **b**, **c**, and **d**, Accumulation patterns of phenolic acid, flavonols, and auxins in *CsUGT84J2-*overexpressing *A. thaliana*, respectively.

To further study the *in vivo* function of *CsUGT84J2 in planta*, its ORF was overexpressed in *A. thaliana*. Over 20 T3 transgenic lines with different expression levels of *CsUGT84J2* transcripts were obtained (Fig. 3a). Corresponding to CsUGT84J2 enzyme activity *in vitro*, changes in phenylpropionic acid, flavonol, auxin, and their corresponding glycosides were analysed using UPLC-QQQ-MS/MS.

Metabolic analysis revealed that compared with those from control lines, the contents of phenylpropionic acids, such as coniferin, syringin, and sinapoylglucose, extracted from *CsUGT84J2*-overexpressing *Arabidopsis* lines were not increased significantly (Fig. 3b). However, the contents of flavonol glycosides were remarkably higher in overexpression lines than in controls, particularly in line 10, in which the contents of the major di-flavonol glycosides, such as K-3-R-7-R, K-3-G-7-R, and Q-3-R-7-R, were increased by over three times (Fig. 3c). In addition to phenylpropionic acid and flavonol glycosides, levels of auxin and its derivatives, such as IAA, IAA-Glc, and IAA-Glu, were increased significantly. In different lines, IAA accumulation was significantly increased, similar to IAA-Glc accumulation (Fig. 3d). Based on these results, *CsUGT84J2* promoted the accumulation of flavonol glycosides and auxins both *in vitro* and *in vivo* (Figs 2 and 3).

### Effects of *CsUGT84J2* on flavonols and auxins in *A. thaliana*

To investigate the effects of *CsUGT84J2* on the metabolism of phenolic acids, flavonols, and auxins, leaves of *CsUGT84J2*-overexpressing *A. thaliana* and *CsUGT84J2-*silenced tea plants were used for transcriptional analysis.

**Figure 4:**
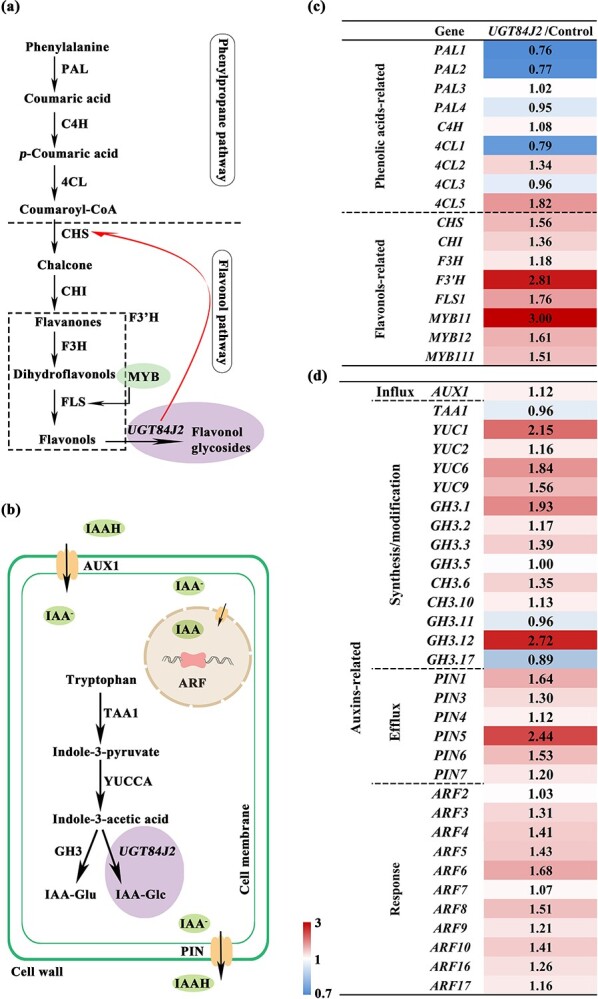
Expression patterns of genes involved in phenolic acid, flavonol, and auxin pathways in *CsUGT84J2*-overexpressing *Arabidopsis thaliana.*

RNA-Seq and qRT-PCR were conducted to examine the expression levels of endogenous genes involved in the phenylpropane, flavonol, and auxin pathways in *CsUGT84J2*-overexpressing and control *A. thaliana* lines (Fig. 4; Fig. S8, see online supplementary material). No significant changes were noted in the expression of phenylpropane pathway genes (Fig. 4a and c). *CsUGT84J2* overexpression increased the expression of most endogenous genes involv0ed in the flavonol pathway, particularly *AtF3′H* and *AtFLS1* (Fig. 4a and c). In addition, the expression of three MYB transcription factors, which control flavonol synthesis, was also upregulated, particularly of *MYB11*, by up to three times (Fig. 4a and c).

Auxin accumulation is affected by its synthesis, modification, influx, and efflux (Fig. 4b). Auxin synthesis is primarily controlled by *TAA* and *YUCCA*. In *CsUGT84J2*-overexpressing *A. thaliana*, *TAA1* expression was slightly altered, whereas four key *YUCCA*s were up-regulated (Fig. 4d). GH3 catalyzes the conversion of auxin to the binding state of the auxin-amino acid conjugate. Notably, expression of *GH3*s, such as *GH3.1* and *GH3.3*, was upregulated in *CsUGT84J2*-overexpressing *A. thaliana*. IAA homeostasis is affected by influx and efflux. Expression of the IAA influx vector (*AUX1*) was slightly increased, and the expression of PIN family genes was increased overall (Fig. 4d; Fig. S7, see online supplementary material). Moreover, auxin response factors (ARFs) were upregulated (Fig. 4d). Synthesis, modification, influx, and efflux of IAA work together to maintain auxin homeostasis in plants. Therefore, high expression of genes related to these key steps jointly enhanced auxin accumulation *in vivo* (Fig. 4b and d).

### Effects of *CsUGT84J2* overexpression on the growth and Al tolerance of *A. thaliana*

**Figure 5:**
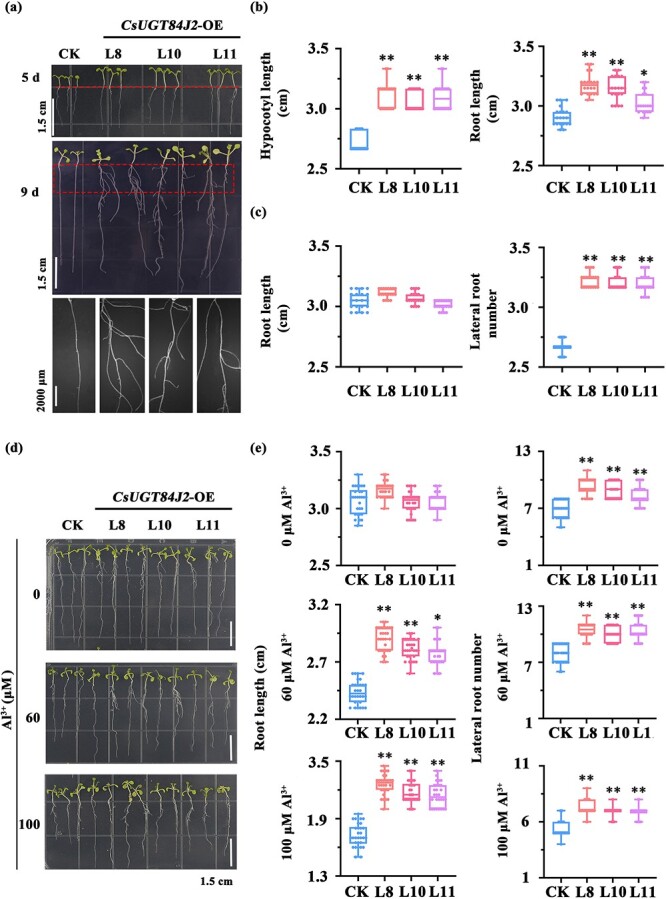
CsUGT84J2 overexpression promoted the growth of and alleviated the Al inhibition in *Arabidopsis thaliana.***a**, Phenotypic analysis of 5- and 9-day-old *CsUGT4J2*-overexpressing *Arabidopsis* seedlings. **b** and **c**, Growth index of 5- and 9-day-old *CsUGT4J2*-overexpressing *Arabidopsis* seedlings, respectively. **d** and **e**, Phenotype and root growth of *CsUGT84J2*-overexpressing *Arabidopsis* plants cultured on solid MS medium supplemented with 0, 60, or 100 μM Al^3+^.

*CsUGT84J2* overexpression affected the gene expression patterns and metabolism of the flavonol and auxin biosynthetic pathways as well as promoted the growth of transgenic *Arabidopsis* plants, particularly of the hypocotyl and lateral roots (Fig. 5). The hypocotyl length of 5-day-old transgenic *A. thaliana* was significantly increased, being approximately two times that of control plants (Fig. 5a and b). In addition, primary root length was increased significantly. With the growth of seedlings, differences in primary root length decreased, whereas the number and length of lateral roots remained significantly higher in *CsUGT84J2-*overexpressing *Arabidopsis* plants (Fig. 5c and d). The lateral root number in 9-day-old transgenic *A. thaliana* plants is three-fold higher than that in control plants (Fig. 5c and d). Overall, in *CsUGT84J2*-overexpressing *Arabidopsis* plants, the upregulated expression of flavonol and auxin pathway genes promoted the accumulation of the related metabolites, resulting in hypocotyl elongation and lateral root development (Figs 3, 4, and 5).

As *CsUGT84J2* is an Al-induced UGT, its overexpression improved the Al tolerance of *A. thaliana*. The physiological phenotypes of *CsUGT84J2*-overexpressing *Arabidopsis* plants cultured on solid MS medium supplemented with low concentrations of Al (0, 60, and 100 μM) were observed and statistically analysed. *CsUGT84J2* overexpression alleviated Al inhibition on *A. thaliana* growth (Fig. 5e and f).

Under 60 or 100 μM Al treatment, the growth of primary roots was inhibited in both control and *CsUGT84J2-*overexpressing *Arabidopsis* lines. However, the inhibition of Al in *CsUGT84J2-*overexpressing lines was significantly weaker than that in control lines (Fig. 5e and f). As mentioned above, *CsUGT84J2* promoted the growth and development of lateral roots in the absence of Al (Fig. 5a and b). Moreover, the number of lateral root in *CsUGT84J2-*overexpressing lines was significantly higher than that in control lines under both 60 and 100 μM Al treatments, although 100 μM Al inhibited *Arabidopsis* growth (Fig. 5e and f). Therefore, *CsUGT84J2* could alleviate the Al-induced growth inhibition in *A. thaliana*.

### *CsUGT84J2* overexpression and suppression in tea plants

**Figure 6:**
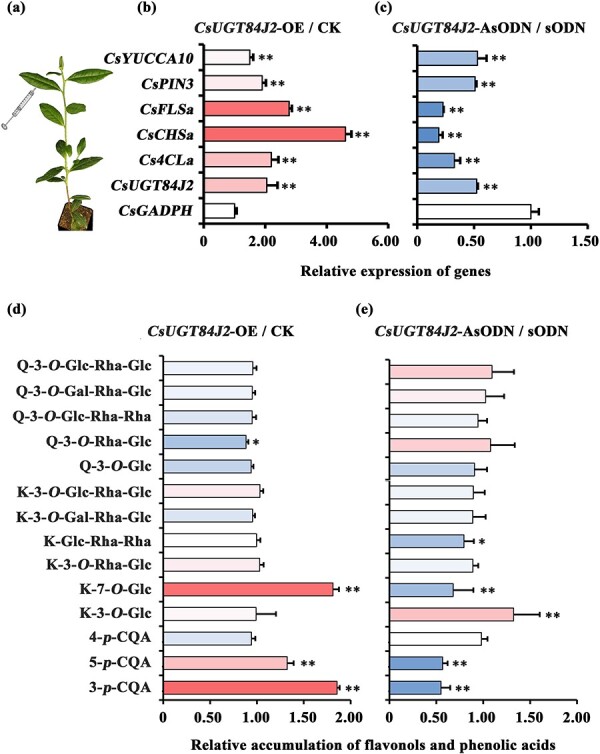
Gene expression and metabolic accumulation related to phenolic acids, flavonols, and auxins in *CsUGT84J2-*overexpressing and *CsUGT84J2-*silenced in tea plants. **a**, Schematic diagram of *CsUGT84J2* overexpression or suppression in tea plants. **b** and **c**, Expression pattern of Cs*UGT84J2*, flavonol-related, and auxin-related genes in *CsUGT84J2*-overexpressing and *CsUGT84J2-*silenced tea leaves, respectively. **d** and **e**, Accumulation patterns of flavonols and phenolic acids in *CsUGT84J2*-overexpressing and *CsUGT84J2-*silenced tea leaves, respectively.

Because of the lack of a stable system of genetic transformation, transient expression was used to study the function of CsUGT84J2 in tea plants via both overexpression and suppression (Fig. 6). qRT-PCR analysis showed that in the leaves of Cs*UGT84J2*-overexpressing and Cs*UGT84J2*-silenced tea plants, Cs*UGT84J2* expression was significantly up- or downregulated, respectively (Fig. 6b and c). Meanwhile, the expression of *Cs4CLa* in the phenylpropane pathway, *CsCHSa* and *CsFLSa* in the flavonol pathway, *CsYUCCA10* and *CsPIN3* in the auxin biosynthesis and efflux pathway were notably upregulated in Cs*UGT84J2*-overexpressing tea leaves, particularly of *CsCHSa* (Fig. 6b). Conversely, in Cs*UGT84J2*-silenced tea leaves, the expression of these genes was significantly downregulated (Fig. 6c).

Subsequently, metabolic changes in phenolic compounds were examined to verify the function of CsUGT84J2 in tea plants. In *CsUGT84J2*-overexpressing tea leaves, the concentrations of K-7-*O*-Glc and *p*-CA derivatives, such as 3-*p*-CQA and 5-*p*-CQA, were significantly enhanced (Fig. 6d). Meanwhile, levels of the other flavonol glycosides did not change significantly. In contrast, in Cs*UGT84J2*-silenced tea leaves, the contents of K-7-*O*-Glc, 3-*p*-CQA, and 5-*p*-CQA were drastically reduced compared with those in Cs*UGT84J2*-overexpressing tea leaves (Fig. 6e).

In conclusion, gene expression and metabolic accumulation related to phenolic acids, flavonols, and auxins were upregulated in *CsUGT84J2*-overexpressing tea plants but downregulated in *CsUGT84J2*-silenced tea plants. Therefore, Al could promote the activity of rCsUGT84J2, thereby enhancing the accumulation of flavonol glycosides and auxin and subsequently promoting the growth of tea plants (Fig. S9, see online supplementary material).

## Discussion

### UGTs affect plant growth via disrupting auxin homeostasis

UGT is a large family, and proteins belonging to the L group of the glycosyltransferases have been closely linked to benzoates [26, 36]. This group of UGTs can be divided into the UGT74, UGT75, and UGT84 subfamilies. Many recombinant proteins of this group of UGTs are involved in auxin glycosylation [13, 28–30]. Auxin glycosylation can store free auxin in the bound forms and is involved in its polar transport. In other words, auxin is transported from the synthetic to other parts and then released [37–40]. Auxin glycosylation products and catabolites are inactive, and they are involved in the regulation of auxin homeostasis and response mechanism [39].

For instance, AtUGT84B1 in *A. thaliana* can catalyze IAA glycosylation, and increase auxin levels in plants, thereby inhibiting the growth of *UGT84B1*-overexpressing plants. While AtUGT74D1 in *A. thaliana* catalyzes oxIAA (an IAA catabolite) glycosylation, it markedly enhanced OxIAA-Glc levels in plants, leading to the loss of root gravitropism [13, 25, 26, 30, 37, 40]. In addition, OsIAGT1 catalyzes the glucosylation of auxin to synthesize IAA-Glc, leading to the reduced root length and plant stature in *OsIAGT1* overexpression rice [41]. Overall, many L group UGTs are involved in the modulation of auxin homeostasis in plants.

In the present study, we screened a tea plant UGT (*CsUGT84J2*), which catalyzes the glycosylation of IAA and IBA *in vitro*. In *CsUGT84J2*-overexpressing *Arabidopsis*, the accumulation of both IAA and IAA-Glc was significantly increased and plant growth was promoted (Figs 3 and 5). Generally, the glycosylation of plant hormones can result in the reduction of free forms of hormones. In our work, we found that the glycosylation of IAA caused the increased IAA concentration in plants. Meanwhile, the expression of genes involved in auxin synthesis and transport was up-regulated (Fig. 4d). We hypothesized that the overexpression of *CsUGT84J2* activated the auxin signaling pathway in plants and restored the accumulation of auxin to a higher homeostasis, involving higher concentrations of free IAA and IAA-Glc.

### Flavonols inhibit plant growth by modulating auxin transport

Contrary to previous reports, CsUGT84J2 was a unique protein in the present study, which could glycosylate both flavonols and auxins. Simultaneously, the recombinant protein showed glycosylation activity on phenolic acid (Fig. 2). Furthermore, our genetic transformation experiments showed that *CsUGT84J2* overexpression increased the content of flavonol glycosides (K-3-R-7-R, K-3-G-7-R, and Q-3-R-7-G) and auxins (IAA and IAA-Glc) in *Arabidopsis* (Fig. 3). Transient expression experiments showed that K-7-Glc, 3-*p*-CQA, and 5-*p*-CQA concentrations were enhanced in *CsUGT84J2*-overexpressing tea plants but downregulated in *CsUGT84J2*-silenced tea plants. Therefore, CsUGT84J2 can glycosylate diverse substrates in plants. Meanwhile, CsUGT84J2 is selective for the hydroxyl sites of flavonols. We previously reported that UGT78 family proteins are specifically glycosylated at 3-OH [27]. Meanwhile, CsUGT84J2 primarily exhibits glycosylation activity on the 7-OH of the flavonol A-ring. Levels of K-3-R-7-R, K-3-G-7-R, and Q-3-R-7-G in *Arabidopsis* and K-7-Glc in tea leaves were significantly increased.

Previous studies have shown that flavonols can affect plant growth and development by interfering with auxin polar transport. Furthermore, under water stress, the flavonoid biosynthetic pathway can be effectively modulated to enhance plant root growth [42]. In addition, flavonols inhibit polar auxin transport by stabilizing the PIN efflux complexes [18]. Kaempferol 3-*O*-rhamnoside-7-*O*-rhamnoside is an endogenous flavonol inhibitor of polar auxin transport in *Arabidopsis* shoots [43]. Similarly, 7-rhamnosylated flavonols can modulate auxin homeostasis to affect plant development [44].

Plants express many types of flavonol glycosides. Specifically, 3-*O* mono-glycosides, di-glycosides, and tri-glycosides are abundant in tea leaves, whereas 7-*O* rhamnosylated glycosides are present at low levels [45, 46]. Meanwhile, in tea roots, rhamnosylated flavonols are accumulated at higher levels than those in leaves (data not shown). Flavonol glycosides involved in IAA regulation remain unclear. In transgenic *Arabidopsis*, kaempferol 3-*O*-rhamnoside-7-*O*-rhamnoside levels were 2.7-fold higher than those in controls (Fig. 3), which may explain the increased Al tolerance of transgenic plants.

Our biochemical and genetic characterization data indicate that the two different types of products catalyzed by CsUGT84J2 are involved in the regulation of the same physiological activity: auxin homeostasis. Flavonols are seen as transport modulators of auxin. Therefore, in tea plants, CsUGT84J2 either enhances flavonol accumulation, influencing auxins concentration indirectly or mediates auxin glycosylation, modulating endogenous auxin homeostasis directly, thereby promoting the growth of tea plants. Nonetheless, the precise mechanisms warrant further investigation.

### Al promotes the growth of tea plant through *CsUGT84J2* induction

In many countries and regions in the world, the soil is mostly acidic and rich in Al ions, which are toxic to many crops. Tea plants are typically Al-tolerant, and low-concentration Al promotes their growth. However, the mechanism through which Al promotes plant growth remains unclear. Al may affect auxin accumulation by regulating its biosynthesis or transport, thus inhibiting the growth of plants such as *A. thaliana*, *Medicago sativa*, and *Zea mays* [2, 47–50]. Whether the effects of Al on the growth of tea plants are related to auxin remains unknown. In the present study, we screened an Al-induced UGT that could glycosylate flavonols and auxins both *in vitro* and *in vivo*, thus affecting auxin homeostasis and promoting the growth of tea plants (Fig. 7). In *CsUGT84J2*-overexpressing *Arabidopsis*, the length of hypocotyls and number of secondary roots increased (Fig. 5). In addition, *CsUGT84J2* overexpression alleviated the Al-induced growth inhibition of *A. thaliana* (Fig. 5).

Two UGTs were identified in the UGT84 subgroup of tea plants. *CsUGT84A22* was mainly expressed in the shoots of tea plants, and CsUGT84A22 exhibited catalytic activity on phenylpropionic and benzoic acid derivatives, particularly gallic acid. The product of gallic acid is β-glucogallin, which is involved in the galloylation of catechins, important phenolics in tea plants [27], or hydrolysable tannins in *Eucalyptus camaldulensis* [51]. Both gallated catechins and hydrolyzed tannins, such as EGCG [8], β-glucogallin, and oenothein B [52] produce Al detoxification effects in plants. In addition to Al detoxification, gallated catechins are the dominant phenolics in tea leaves, which determine the flavor of tea, particularly astringency [46].

*CsUGT84J2* was mainly expressed in tea roots. CsUGT84J2 acted on three types of substrates, including phenolic acids, flavonols, and auxins, *in vitro* (Fig. 2 and Table 1). In *CsUGT84J2*-overexpressing *A. thaliana*, flavonol and auxin accumulation was significantly increased (Fig. 3), which was closely related to the growth and development of tea plants. Moreover, the expression of flavonol and auxin-related genes was upregulated in *CsUGT84J2*-overexpressing *Arabidopsis* and tea plants but downregulated in *CsUGT84J2*-silenced tea plants (Figs 4 and 6). In other words, Al-induced *CsUGT84J2* promoted the expression of flavonol and auxin pathway-related genes and accumulation of the related compounds, thus promoting the growth of tea plants (Fig. 6 and Fig. S9). However, the mechanism that Al-induced *CsUGT84J2* mediates flavonol and auxin glycosylation to promote the growth of tea plants requires further research. As such, whether auxins or flavonols play a dominant role, whether the two complement each other, or whether they act synergistically remains obscure.

## Materials and methods

### Al treatment of tea plants

Leaves and young roots at different developmental stages or young roots treated with Al were collected from *Camellia sinensis* ‘Shuchazao’ growing in the tea plantation of the Anhui Agricultural University (Hefei, Anhui Province, China; 31.52°N, 117.14°E). The samples were snap frozen in liquid nitrogen and stored in a refrigerator at −80°C for subsequent experiments.

For Al treatment in tea plantation, 5-year-old ‘Shuchazao’ plants from the experimental tea plantation of the Anhui Agricultural University were treated with 2 mM Al^3+^ for 6 months, followed by treatment every 2 weeks, from July to December 2020, using 2 L of Al solution each time. AlCl_3_·6H_2_O were purchased from Sinopharm Chemical Reagent Co., Ltd (Shanghai, China).

### Cloning and expression of *CsUGT84J2* in *E. coli* and its enzyme activity assay

Total RNA was extracted from the aforementioned organs of tea plants using an ultrapure RNA kit (Vazyme Biotech Co., Ltd, Nanjing, China) following the manufacturer’s protocol. Primers based on the open reading frame (ORF) of *CsUGT84J2* were used and are listed in Table S1 (see online supplementary material).

The ORF of *CsUGT84J2* was constructed into the expression vector pMAL-c2X with a maltose-binding protein using an *in vitro* enzyme digestion method. The restriction sites of the expression vector were BamHI and SalI, and the primers used are listed in Table S1 (see online supplementary material). The expression vector (pMAL-CsUGT84J2) with the target gene was then transferred into the expression host strain BL21. The protein purification method proposed by Dai *et al.* [31] was used. Coomassie brilliant blue was used to measure the protein concentration using a spectrophotometer, and a 12% sodium dodecyl sulphate–polyacrylamide gel was used to analyse the purified CsUGT84J2 recombinant protein.

In the enzymatic assays and kinetic analysis of the recombinant rCsUGT84J2 protein, coumaric acid, kaempferol, and IAA were used as sugar acceptors and UDP-glucose or UDP-rhamnose were used as sugar donors to determine substrate activity. UDP-glucose was selected as the sugar donor to analyse various factors affecting enzyme activity. Enzyme activity was calculated based on increase in the product peak area after the reaction. The test factors included temperature, buffer, and pH. All reaction mixes (50 μL) contained 20 μg of recombinant rCsUGT84J2; 2 mM UDP-glucose; and 0.5 mM kaempferol, *p*-coumaric acid, or IAA, raised to 50 μL with buffer. For the temperature test, 50 mM Tris–HCl (pH 7.5) was added, and the reaction was conducted at five different temperatures (20°C, 30°C, 35°C, 40°C, and 45°C). For the buffer and pH tests, 100 mM citric acid–sodium citrate (pH 5.0–7.0), 50 mM Tris–HCl (pH 7.0–9.0), or 50 mM glycine–NaOH (pH 9.0–11.0) buffer were added, and the reaction was conducted at 30°C or 40°C. All reactions lasted for 90 min and were stopped by adding 50 μL of chromatographic methanol. The reaction systems were snap frozen and stored at −20°C before reverse-phase high-performance liquid chromatography (HPLC) analysis. HPLC (Agilent Technologies, Palo Alto, CA, USA) conditions are presented in Table S2 (see online supplementary material). The standard products of the above compounds were purchased from Shanghai Yuanye Bio-Technology Co., Ltd (Shanghai, China). Tris–HCl, citric acid, sodium citrate, and glycine were purchased from Sinopharm Chemical Reagent Co., Ltd (Shanghai, China).

### Plasmid construction and heterologous expression of *CsUGT84J2* in *A. thaliana*

Primer sequence with the attb linker was ligated to the complete CsUGT84J2 ORF using the *in vitro* PCR amplification technique. The PCR product was purified using the Gateway BP cloning enzyme mixture, cloned into the entry vector pDONR207 (Lab of Xiangchengbin, USTC), and then into the expression vector pCB2004 (Lab of Xiangchengbin, USTC, Heifei, China) using the Gateway LR cloning enzyme system. The expression vector with the target gene (pCB2004-CsUGT84J2) was transferred into the expression host strain GV3101 (Lab of Xiangchengbin, USTC, Heifei, China). The primer sequences are listed in Table S1 (see online supplementary material).

The recombinant pCB2004-CsUGT84J2 plasmid was chemically transformed into GV3101. Wild-type *Arabidopsis* (Col-0) was used for *Agrobacterium tumefaciens*-mediated transformation.

### Extraction and quantification of phenolics and auxin

The ultra-performance liquid chromatographic (UPLC) separation method summarized in Table S2 (see online supplementary material) was used to systematically analyse the products obtained from the enzyme activity assays and kinetic analysis. Phenolic acid and flavonol glycosides were extracted from the leaves of 21-day-old *Arabidopsis* seedlings and analysed according to a previously described method [45]. Moreover, flavonol glycosides were extracted from the roots of tea plants treated with Al for 6 months and analysed according to a previously described method [46]. The conditions for UPLC and MS/MS analysis are summarized in Table S3. Relative quantification was based on the area of the major MS/MS signals (M-H)^+^.

For quantification of the amide conjugates IAA and IAGlc, approximately 200 mg samples of whole plant tissue (21-day-old) were used. After extraction with 1 mL of ethyl acetate for 10 min at 25°C in a ball mill, the samples were centrifuged. Then, organic solvent in the supernatant was removed *in vacuo*, the precipitate was dissolved in methanol, diluted with water two times, and analysed with UPLC-QqQ-MS/MS (Agilent Technologies, Palo Alto, CA, USA). The conditions for UPLC and MS/MS analysis are summarized in Table S4 (see online supplementary material). IAA, IAA-Asp, IAA-Glu, and IAA-Glc were analysed as previously reported [13]. Relative quantification was based on the area of the major MS/MS signals (M-H)^+^.

### RNA-Seq and qRT-PCR

To examine the expression patterns of genes related to the phenylpropanoid, flavonoid, and auxin-related pathways in *Arabidopsis* overexpression lines, RNA-Seq was performed by BGI (Wuhan, China) on 21-day-old seedlings. Three biological replicates of *CsUGT84J2* overexpression and control groups were used. A nucleic acid quantifier (NANODROP 1000, Thermo Scientific, Massachusetts, USA) was used to quantify the RNA extracted from various tissues and organs of tea plants subjected to Al, gene overexpression, and gene silencing treatment as well as that extracted from *Arabidopsis* plants.

Using 5× PrimeScript RT Master Mix (Takara), the RNA was reverse transcribed into cDNA, and qRT-PCR was performed using a previously proposed method [53]. The primers for qRT-PCR are listed in Table S1 (see online supplementary material).

### *CsUGT84J2* suppression in tea plants

Candidate antisense oligonucleotides (AsODNs) were selected using the SOLIGO software, with *CsUGT84J2* as the input sequence. Three pairs of sequences that met the requirements were screened, and their reverse non-complementary sequences were used as controls (Table S1, see online supplementary material). AsODNs were synthesized by General Biosystems. To silence *CsUGT84J2* in tea leaves, 1 mL of 20 μΜ Cs*UGT84J2* AsODN solution was injected into the mature leaves of tea cuttings; leaves injected with sense oligonucleotides (sODNs) were used as controls [54]. After 72 h of incubation, the samples were snap frozen in liquid nitrogen for gene expression and metabolism analysis.

### *CsUGT84J2* overexpression in tea plants

*CsUGT84J2* was inserted into the pMCABIA1305.1-GFP vector and transferred to the host strain GV3101; pMCABIA1305.1-GFP empty vector was used as the control. Then, the bacterial solution was washed, re-suspended in MES suspension, and injected into the mature leaves of tea cuttings. After 72 h of incubation, the samples were frozen in liquid nitrogen for gene expression and metabolism analysis. At least three biological replicates were included.

### Al treatment of *A. thaliana* seedlings

For experiments on *Arabidopsis*, the seeds were surface-disinfected with 25% concentration of 84 disinfectants for 3 min and then washed three to four times with sterile deionized water. The seeds of three lines overexpressing *CsUGT84J2* and controls were sown in disposable Petri dishes (diameter = 90 mm) containing solid MS medium and incubated under a 16/8 h light/dark cycle at 20 ± 2°C for 5 or 9 days. The primary root length, hypocotyl length, and lateral root number of *CsUGT84J2-*overexpressing and control seedlings were determined.

Following Al treatment, 5-day-old seedlings were carefully removed from the MS sucrose agar plates, placed on solid MS medium supplemented with Al^3+^ (0, 60, and 100 μΜ), and incubated at 20 ± 2°C under continuous light for 5 days. Then, root length and lateral root number under different Al treatments were measured. The biological experiment was repeated three times, with 60 seedlings treated each time.

### Statistical analysis

The biological duplicate data were analysed statistically by one-way ANOVA using the software IBM SPSS Statistics V21.0, and the means were compared for significant differences by Duncan analysis; a *P* value ≤0.05 was considered significant.

## Acknowledgments

This work was supported by the Natural Science Foundation of China (31902069 and U21A2023), the Youth Science and Technology Talents Support Program (2020) by Anhui Association for Science and Technology (RCTJ202010), the College Students' Innovative Training Program of Anhui Province (S202110364265) and the Collegiate Collaborative Innovation Foundation of Anhui Province (GXXT-2020-081).

## Author contributions

The presented study was conducted in collaboration by all authors. X.J, S.L., L.G., and T.X. conceived and designed the experiments. S.L., X.J., D.K., Y.S., X.H., and Z.F. performed the experiments. S.L., X.J., and Z.F. analysed the data. X.J., S.L., L.G., Y.L., and T.X. wrote the manuscript. All authors reviewed the manuscript.

## Data availability

All relevant data in this study are provided in the article and its supplementary file. The genotypic datasets presented in this study can found at: https://www.ncbi.nlm.nih.gov/, with accession numbers CsUGT84J2 (KP682363.1).

## Conflict of interest statement

The authors declare no competing financial interests.

## Supplementary data

Supplementary data is available at *Horticulture Research* online.

## Supplementary Material

Web_Material_uhad095
