# Supplementary material for: Al-induced CsUGT84J2 enhances flavonol and auxin accumulation to promote root growth in tea plants
Source: Hortic Res. 2023 May 5;10(6):uhad095. doi: 10.1093/hr/uhad095 (PMC10282599; doi:10.1093/hr/uhad095)
Supplement: Web_Material_uhad095 [file web_material_uhad095.zip › Supplementary Figures.docx]

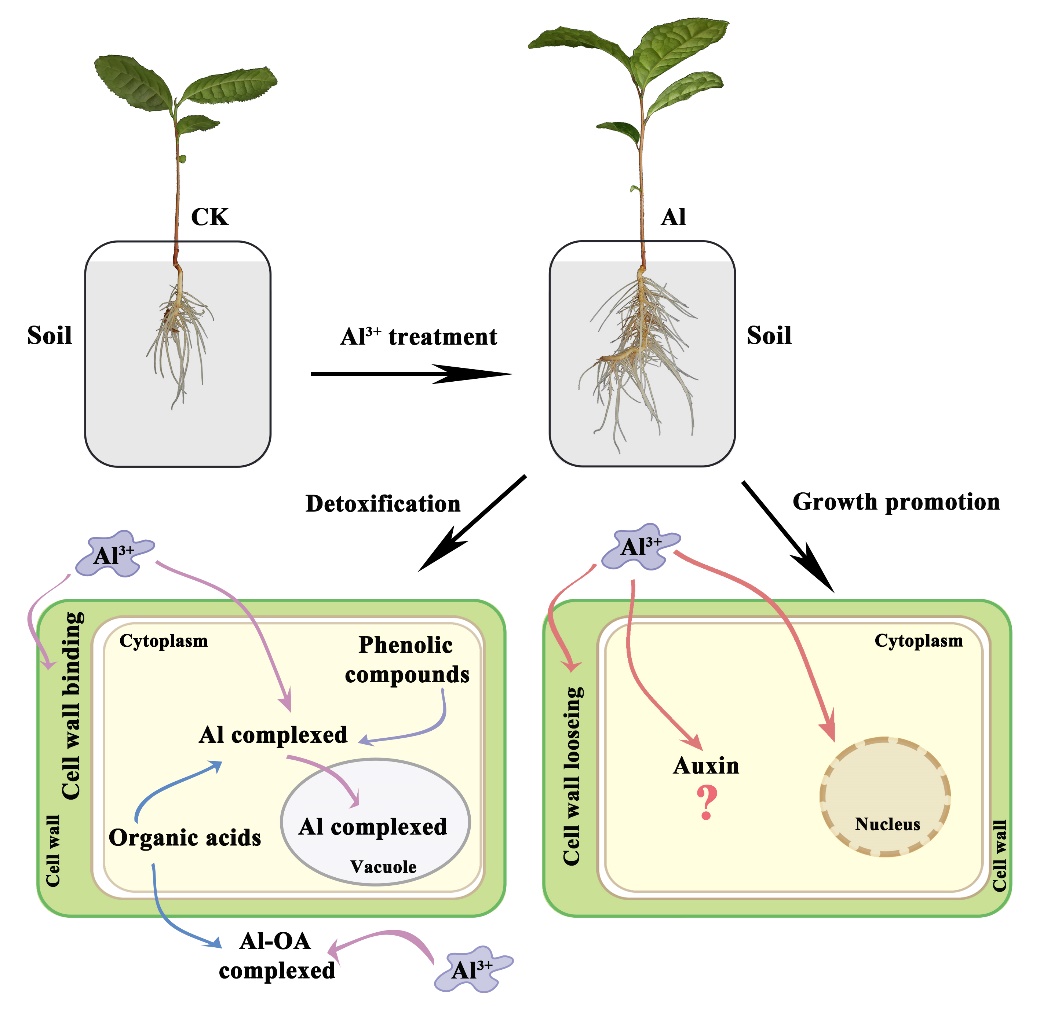


**Fig. S1 Currently understood mechanisms of aluminum tolerance in tea plants.**

**
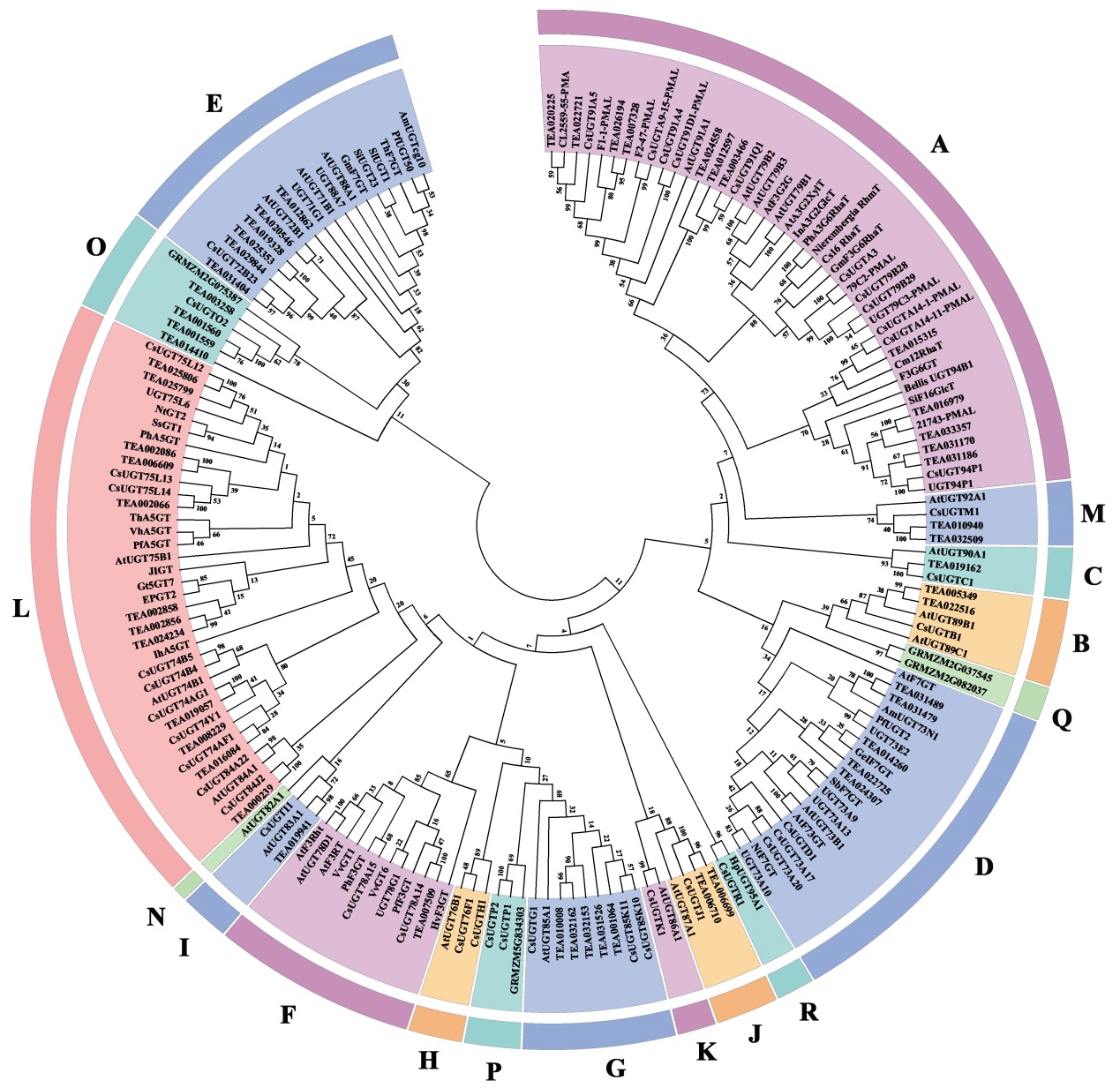
**

**Fig. S2 Phylogenetic tree analysis of UGTs induced by aluminum.**

**
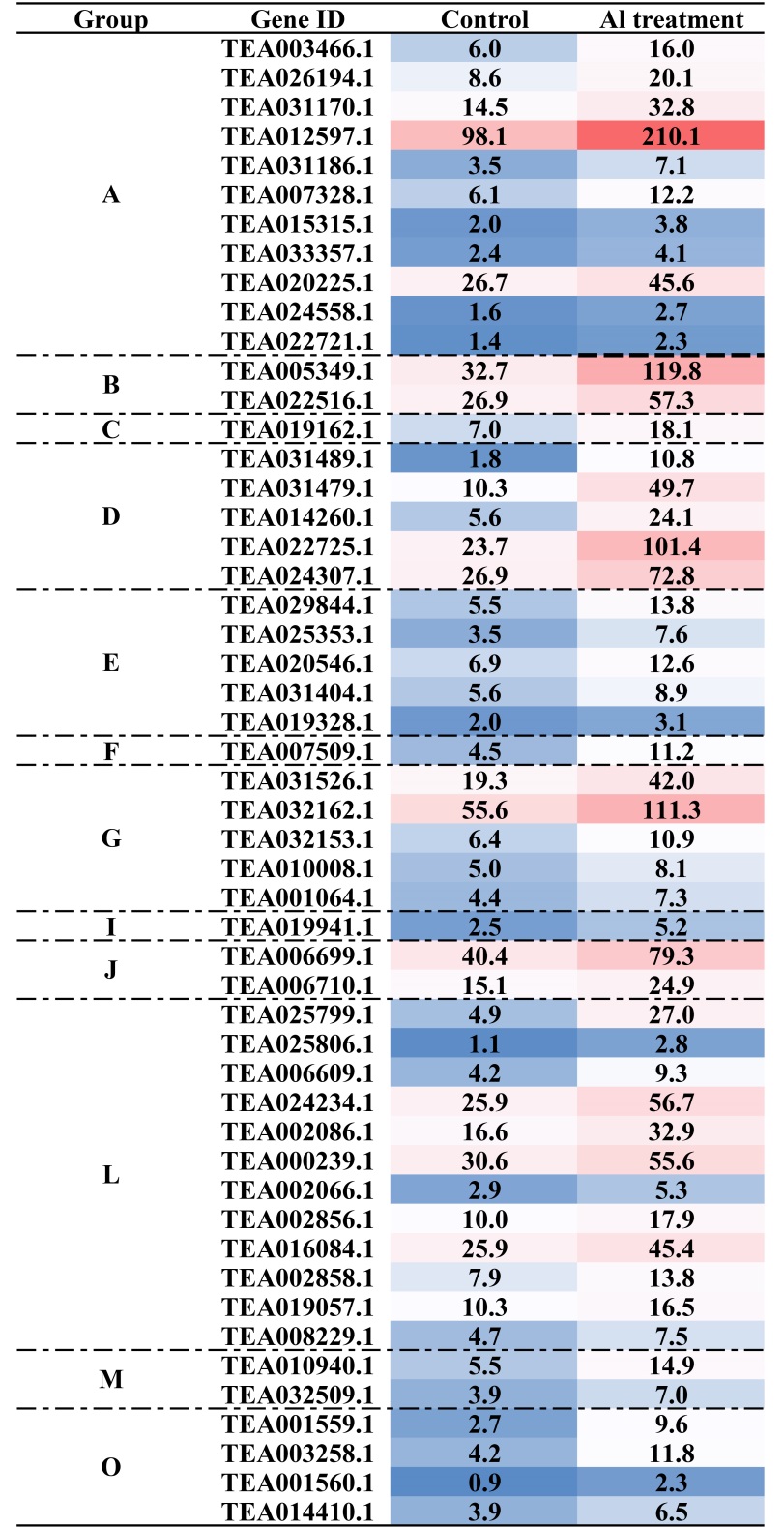
**

**Fig. S3 Gene expression analysis of UGTs induced by aluminum.**

**
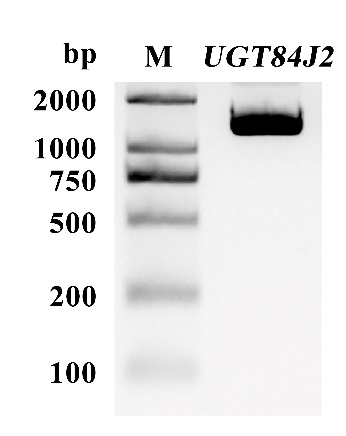
**

**Fig. S4 Agarose gel electrophoresis of *CsUGT84J2***

**
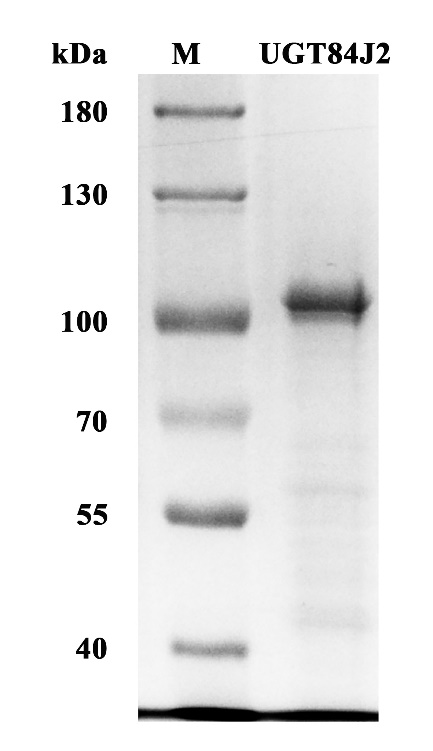
**

**Fig. S5 SDS-PAGE electropherogram of rCsUGT84J2**

**
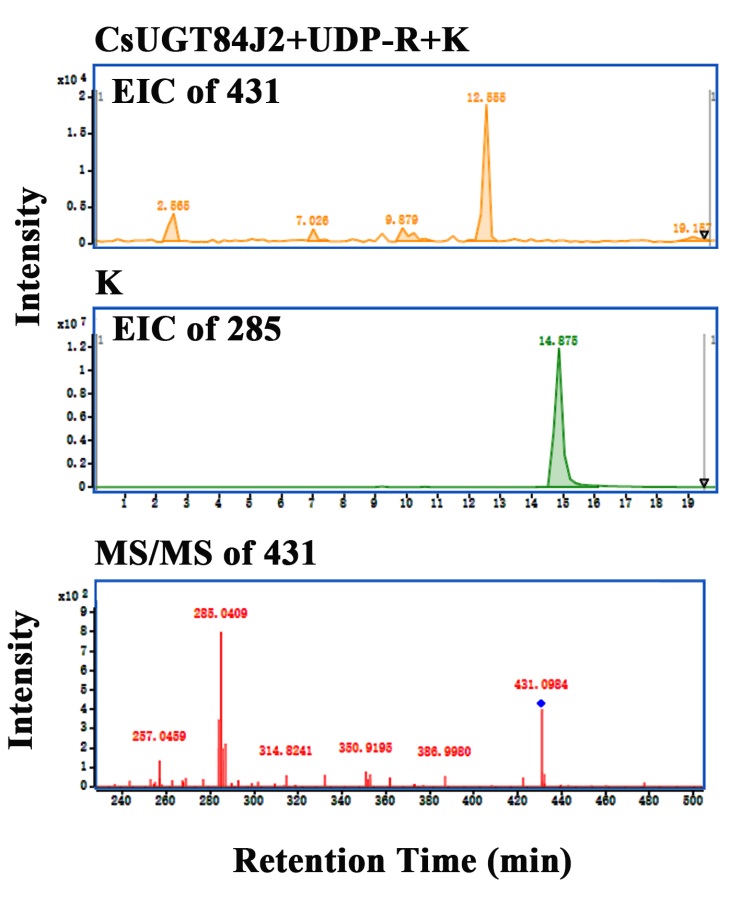
**

**Fig. S6 MS/MS analysis of the enzymatic reaction product kaempferol rhamnoside.**


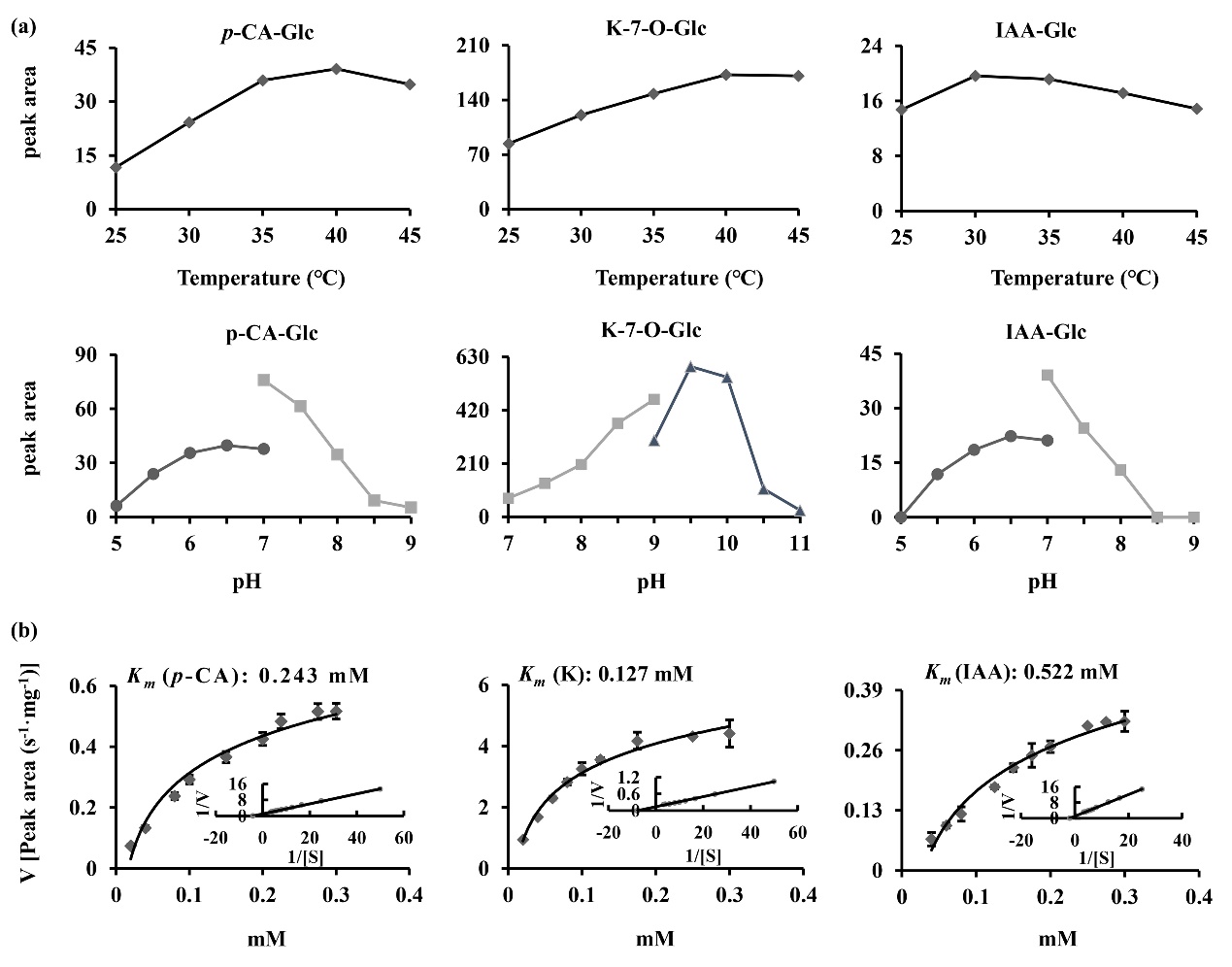


**Fig. S7 Biochemical characterization of rCsUGT84J2 protein using *p*-coumaric acid, kaempferol and indole-3-acetic acid as substrates. a, Optimization of the reaction temperature of rCsUGT84J2 to three substrates; b, Optimization of the reaction pH of rCsUGT84J2 to three substrates.**

**Fig. S8 Expressin pattern of some genes in *CsUGT84J2*-overexpressed *Arabidopsis thaliana* by qRT-PCR verification*.***


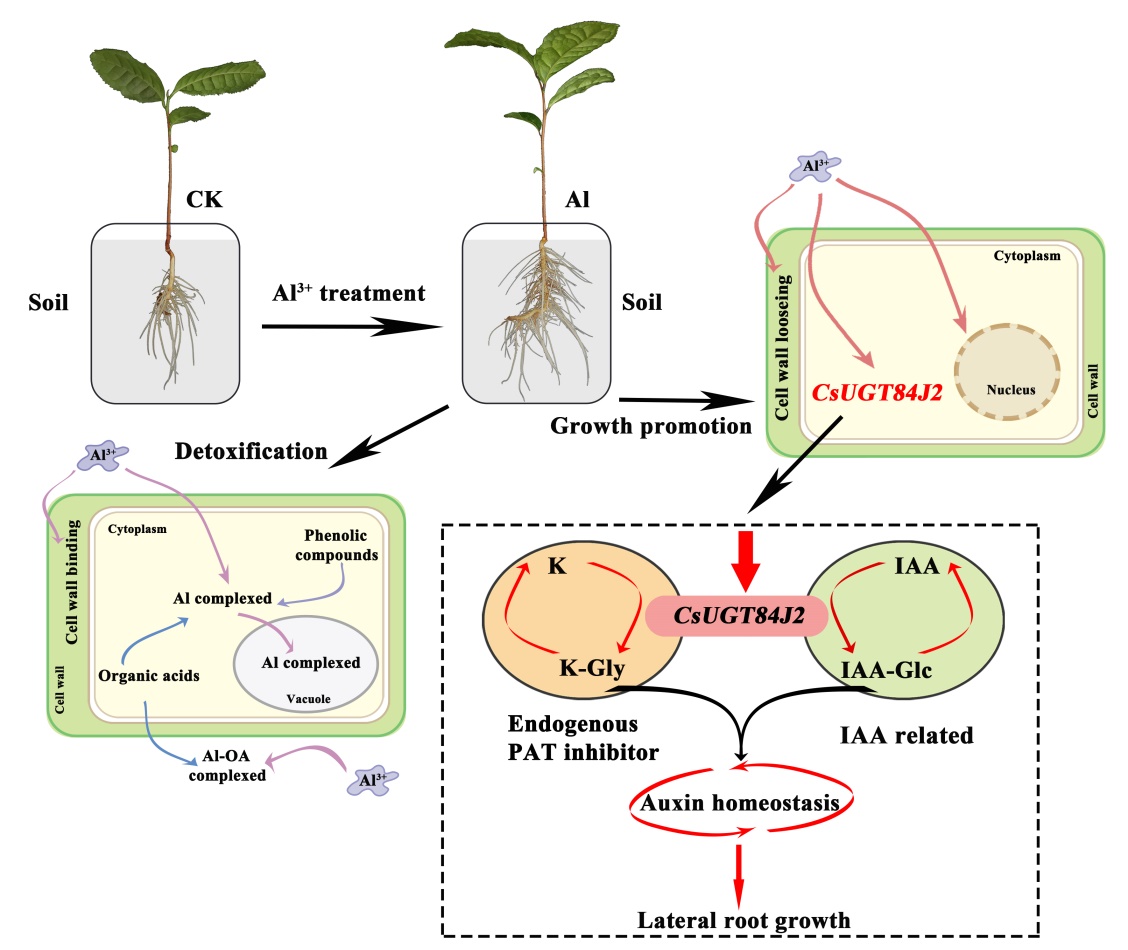


**Fig. S9 Working model of the *CsUGT84J2*-mediated promotion of tea plant growth following Al treatment.**
